# A Reactive Molecular Dynamics Study on Crosslinked Epoxy Resin Decomposition under High Electric Field and Thermal Aging Conditions

**DOI:** 10.3390/polym15030765

**Published:** 2023-02-02

**Authors:** Wei-Feng Sun, Wen Kwang Chern, John Chok You Chan, Zhong Chen

**Affiliations:** 1 SP Group—NTU Joint Laboratory, School of Electrical & Electronic Engineering, Nanyang Technological University, Singapore 639798, Singapore; 2 Singapore Power Group, Singapore 349277, Singapore; 3 School of Materials Science and Engineering, Nanyang Technological University, Singapore 639798, Singapore

**Keywords:** epoxy resin, partial discharge, thermal-electrical synergy, polymer decomposition

## Abstract

To reveal the microscopic mechanism of synergetic thermal–electrical degradation during a partial discharge process in epoxy insulation materials, the decomposition of crosslinked epoxy resin is investigated using reactive molecular dynamics simulations under high electric field and thermal degradation conditions. Bond-boost acceleration method is employed in reactive molecular dynamics simulations to successfully establish epoxy polymer models with a crosslink degree of 93%. Active molecular species derived from electrical partial discharges are considered in the current work. Small molecule products and decomposition temperature in the degradation process under an electric field are calculated to elucidate the effect of nitric acid and ozone molecules, being the active products generated by electrical partial discharges, on the synergetic thermal–electrical degradation of epoxy resin. Both nitric acid and ozone exacerbate thermal impact decomposition of crosslinked epoxy polymer by decreasing initial decomposition temperature from 1050 K to 940 K and 820 K, respectively. It is found that these active products can oxidize hydroxyl groups and carbon–nitrogen bridge bonds in epoxy molecular chains, leading to the aggravation of epoxy resin decomposition, as manifested by the significant increase in the decomposed molecular products. In contrast, thermal degradation of the epoxy resin without the active species is not expedited by increasing electric field. These strongly oxidative molecules are easily reduced to negative ions and able to obtain kinetic energies from electric field, which result in chemical corrosion and local temperature increase to accelerate decomposition of epoxy insulation materials.

## 1. Introduction

Epoxy resin (EP) is a dominant dielectric thermoset polymer material applied in electrical engineering [1,2,3], aerospace, medical, and other electric equipment operating in extremely severe environments [4]. Epoxy resin, as a characteristic dielectric material, has been applied as a dielectric terminal of superconducting electric power equipment due to its excellent mechanical properties, dielectric performances, and chemical stability at low-temperatures [5]. In particular, dielectric EP for the current terminal of superconducting electric equipment can simultaneously sustain a strong electric field and large temperature gradient. Epoxy resin is also a comprehensively used insulating material for cables, winding structures in solid-state transformers, and power electronic devices [6]. High-voltage dielectric materials are always under risks of partial discharge and accelerated degradation due to the combined thermal and electrical stresses. Accordingly, the casting epoxy polymers are generally utilized for power electronic devices and insulators to withstand insulation failure under high electric and thermal stresses [7]. On the other hand, epoxy resin nanocomposites, in which inorganic or metallic nanofillers are dispersed uniformly into epoxy resin matrix, have recently attracted great attention due to their prospective potentials in dielectrics and electrical insulation [8,9,10].

Partial discharge dominates the electrical deterioration of polymer insulation materials by initiating carbonized conductance channels and resulting in full electrical breakdown. Scattered impact, chemical corrosion, and thermal effect of dielectric surface under a high electric field will decrease transient dielectric strength and expedite the degradation process of polymer insulation materials [11]. The accumulation of partial discharges results in electric-tree aging, which will eventually develop into the electric breakdown of polymer insulation materials [12]. In the operation of electric equipment, polymer insulation materials suffer diverse unfavorable loads, such as electricity, heat, mechanical stresses, and chemical corrosion, which can potentially accelerate the degradation process. The mechanism of insulation degradation caused by partial discharge is very complex, and the general understanding of insulation degradation includes charge bombardment, thermal effect, the chemical corrosion of active by-products, high-energy radiation, and mechanical stress. The first three factors play a major role in the degradation of polymer insulation materials. It is generally believed that a larger temperature gradient leads to a lower partial discharge inception voltage. Depending on the power device design, a large temperature gradient and high-stress electric field can exist in certain critical locations that make the device vulnerable to partial discharge. Therefore, it is of great significance to study the degradation mechanism of EP polymers used in electric power equipment under partial discharges. Such a study could help improve the long-term reliability of electric power equipment through improved material designs.

At present, insulation performances of polymer materials are studied primarily by characterizing the dielectric state after degradation, such as the thermal decomposition characteristics and the final products [13,14,15,16]. Using a needle-plate test structure under a sinusoidal voltage pulse with an exponentially decaying amplitude, Gui et al. found that partial discharge is more likely to be initiated under a higher frequency or a lower magnitude of sinusoidal pulse voltage [17]. The aspect ratio of carbon nanotubes and their dispersion in EP matrix can be appreciably improved by the organic functionalization of grafting mixed acid, without any notable increment in thermal conductivity [18]. However, not all experimental and analytical calculation approaches can be exploited to monitor the polymer decomposition process in real time to elucidate the microscopic mechanism of dielectric aging of insulation materials.

Reactive force field (ReaxFF) has been used to simulate the thermal decomposition process of noncrosslinked EP copolymers and analyze the chemical reaction path for small molecule products at various temperatures [19,20]. Previous studies have successfully employed reactive molecular dynamics (MD) simulations on reaction rates, species products, and initiation time of polymer decomposition and organic compound combustion at high-pressure and -temperature conditions with an extremely strong electrostatic field that cannot be characterized by high-voltage experiments such as dielectric breakdown and partial discharge for polymeric insulation materials [21,22,23]. Reactive MD simulations have revealed the intrinsic mechanism and chemical reaction path of small molecule products such as water and hydrogen, and proved that the thermal decomposition of EP polymers is initiated by breaking oxygen bridge bonds adjoining to benzene rings [24,25,26,27]. Synergetic electro-thermal decomposition of carbon nanotube/epoxy composites was revealed by reactive MD simulations, and the unsealed carbon nanotubes were found to be effective in improving the thermal stability by inhibiting molecular-chain thermal motions in EP polymers [25]. MD simulations adopting ReaxFF can also be implemented to evaluate the impacting damages of atomic oxygen bombardments by calculating mass loss, molecular product, transient temperature, and surface penetration depth [26]. ReaxFF is also capable of simulating erosion-resistant characteristics of spacecraft polymeric materials under impacts of high-velocity ions [27]. Therefore, ReaxFF should be capable of simulating the electro-thermal decomposition of crosslinked EP polymers under electrical impacts of active ions that are generated by surface electrical creepage discharge or electrical partial discharge.

In the present study, the bond-boost acceleration method is used in reactive MD simulations to construct an epoxy polymer model with a crosslink degree higher than 90%. To reveal the reaction pathways, species products, and initial temperature of EP polymer decomposition under externally applied electrostatic fields and thermal impact, we utilize a ReaxFF to perform heat-rating MD simulations under high electrostatic field for EP polymers with the chemically active impurities as produced by partial discharges. In combination with first-principles calculations, the effects of nitric acid and ozone molecules as the two paradigms of partial-discharge-produced active impurities on the initial temperature and molecular products of EP polymer decomposition under high electrostatic fields and thermal-impacts are investigated to elucidate the molecular-level mechanism of EP degradation under partial discharges.

## 2. Reactive Molecular Dynamics Simulation

### 2.1. Crosslinked Model of Epoxy Polymer

**Figure 1:**
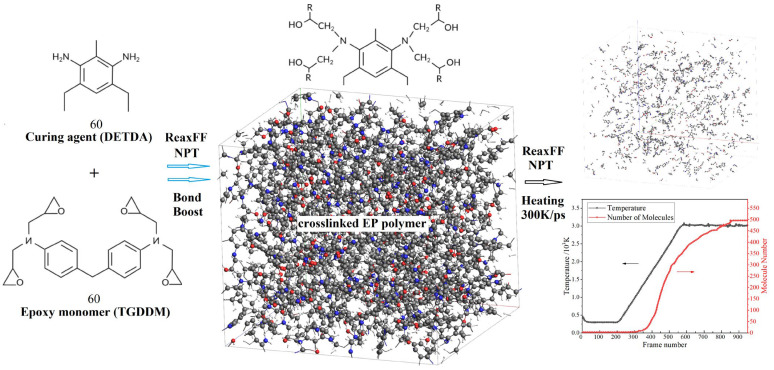
Schematic process of modeling crosslinked EP polymers, in which the gray, blue, red, and white spheres denote carbon, nitrogen, oxygen, and hydrogen atoms, respectively.

Noncrosslinked (noncured) and crosslinked (cured) EP materials are different in their properties. In the current work, crosslinked network model of cured EP polymer is firstly established by crosslinking N,N’-tetraglycidyl diaminodiphenylmethane (TGDDM) polymerization monomer and diethyltoluenediamine (DETDA) curing agent, as shown in Figure 1. By packing 60 TGDDM monomers and 60 DETDA curing agent molecules into a periodic boundary amorphous unit cell with an initial density of 0.61 g/cm^3^, which is approximately half of the cured EP materials. Bond-boost acceleration method of AMS ReaxFF module is employed to generate the highly crosslinked structure (realistic polymer model) of EP polymers, in which dispersion reactive force field (CHONSSi-lg.ff) is adopted to perform MD simulations under isothermal–isobaric (NPT) ensemble [28,29].

Total simulation time, target thermodynamic temperature, and pressure are specified as 100 ps, 500 K, and 0.1013 MPa, respectively, under which the actual curing reactions of epoxy monomers generally occur in experimental preparations of crosslinked epoxy resin materials, to obtain a crosslinked model of EP polymer with a crosslink degree of 93%. The 100 ps total time of reactive MD simulations with bond-boost schemes have been verified by us eligible for constructing the cured epoxy polymers with a crosslink degree higher than 90%. Subsequently, 50 ps NPT reactive MD simulation at ambient condition (298 K, 0.1013 MPa) is performed to obtain the crosslinked EP polymer model with density of 1.12 g/cm^3^. This model is used for reactive MD simulations to investigate EP polymer decomposition under high electric fields or thermal aging conditions. It should be noted that the polymer models and reactive MD simulations in current work are conforming to ideal kinetics without incorporating the competition of intra- and intermolecular reactions [30].

Thermal electrons excited by electrical partial discharge may collide with the ambient nitrogen, oxygen, and water molecules, which could produce multiple active products mainly comprised of ozone and nitric acid. These active molecules are oxidative and can cause local high temperatures by impacting with molecular chains of polymer insulation materials under a high electric field, which will expedite the chemical decomposition. In this work, molecular models of nitric acid and ozone (HNO_3_ and O_3_) are constructed by first-principles geometrical optimization [31], which are then packed into the amorphous cell of crosslinked EP polymer model. Eventually, reactive MD simulations of NPT ensemble under ambient condition are performed for 50 ps to build the impurity-containing model of EP polymer.

### 2.2. Reactive MD Simulation Schemes

Reactive MD simulations of NVT and NPT ensembles are carried out by HCONSB.ff reactive force field for pure and impurity-containing crosslinked EP polymer, as implemented by ReaxFF code of Amsterdam Modeling Suite (AMS) software package. The HCONSB.ff reactive force field is specified for describing chemical reactions of the molecules with C, H, O, or N bonding atoms. The distorted electric field that initiates electrical partial discharge in insulation materials used for high-voltage power system will reach hundreds of kV/mm, and the electrical partial discharge can promptly increase the local temperature to thousands of Kelvin. In addition, it has been demonstrated by reactive MD simulations that a higher specified value of simulation temperature and heating rate result in a higher speed and a lower initial temperature of polymer thermal decomposition, respectively, while neither of them evidently account for product species and decomposition reaction path [20,32,33]. To elucidate the chemical corrosion under extremely high electric field of partial discharges, the active molecules generally produced by partial discharges are modeled and added into the crosslinked EP models to perform NVT MD simulations for 50 ps under 0.001~0.01 V/Å electrostatic fields, in which the sharp temperature increasing (heating) rate is adopted to simulate thermal effect of partial discharges. To simulate thermal-impacted decomposition of EP polymers caused by thermal effects of partial discharges, the heating-rated NPT MD simulations for 50 ps are performed without applying an electric field. The final highest temperature point of 3000 K and a heating-rate of 300 K/ps, which could be promptly approached in the local regions where partial discharge occurs, are specified for the suddenly heated (temperature boost up) reactive MD simulations to represent the thermal impact caused by partial discharges, elucidating the initial temperature and reaction progress of the chemical decomposition of EP polymeric crosslink network under thermal effect of partial discharge [34].

## 3. Results and Discussion

### 3.1. Decomposition Temperature under Electric Field

**Figure 2:**
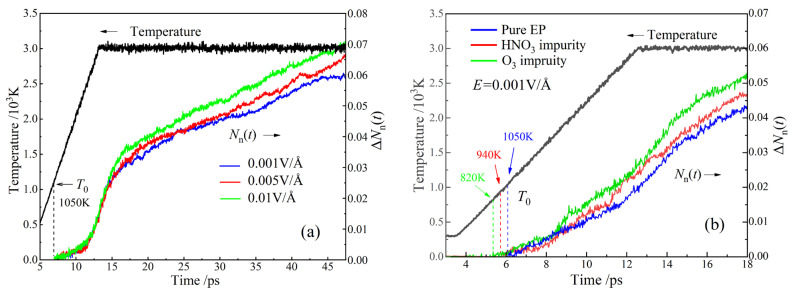
Normalized increment of molecule number with time in reactive MD processes of the crosslinked EP polymer models (**a**) without molecular impurity and (**b**) with active molecular impurities under thermal–electrical synergy (electric field + raised temperature).

Epoxy polymer decomposition during the reactive MD process is evaluated by the normalized increment of molecular number Δ*N*_n_(*t*) = [*N*_molecule_(*t*) − *N*_molecule_(*t*_0_)]/*N*_atom_ as a function of time *t*, as shown in Figure 2a for the pure EP polymer model. *N*_atom_ and *N*_molecule_ denote the total number of atoms and molecules in the condensed matter models of EP polymers, and *t* is the time in the MD simulation. Initial decomposition time *t*_0_, which characterizes the initial decomposition temperature, is independent of the electric field strength applied to the pure epoxy polymer model. In contrast, the rate and magnitude of *N*_n_(*t*) increase with electric field strength at the end heating stage (Figure 2b) when the quasi-thermodynamic temperature approaches 3000 K, implying that the electric field can substantially aggravate thermal-impacted decomposition of EP polymers. A variety of polar molecules and racial groups of decomposition products can acquire a considerable kinetic energy from high electric field, which can accelerate the decomposition process. Therefore, under thermal–electrical synergy, the temperature dominates the decomposition of pure epoxy resin.

Initial time *t*_0_ of EP polymer decomposition that occurs for the three crosslinked models is obviously different, which can be used to identify initial decomposition temperature (*T*_0_) by a heating rate of 300 K/ps during the reactive MD process, as shown in Figure 2. The quasi-thermodynamic equilibrium of NVT ensemble is reached in the heated MD process, as indicated by Δ*N*_n_(0*~t*_0_), which remains zero. The pure EP polymer represents a notably lower Δ*N*_n_(*t*) than the ones containing HNO_3_ or O_3_ impurity when MD time is longer than 9 ps, in which thermodynamic temperature reaches 1900 K.

Both nitric acid and ozone can exacerbate the synergetic thermal–electrical decomposition of EP polymers, as manifested by the increased decomposition products and the reduced initial decomposition temperature. As indicated in Figure 2b, the existing HNO_3_ or O_3_ impurity results in a perceptible reduction in initial decomposition temperature from 1050 K to 940 K, whilst facilitating EP decomposition to engender a larger amount of molecular products, as indicated by Δ*N*_n_(*t*). Comparing the two species, ozone is more powerful of facilitating EP polymer decomposition at temperatures below *T*_0_, and there are more decomposition products at temperatures higher than 1900 K. It is concluded that the active molecular impurities produced by electrical partial discharge will significantly reduce the thermal stability of EP materials under high electric fields.

### 3.2. Thermal Impact Decomposition without Electric Field

**Figure 3:**
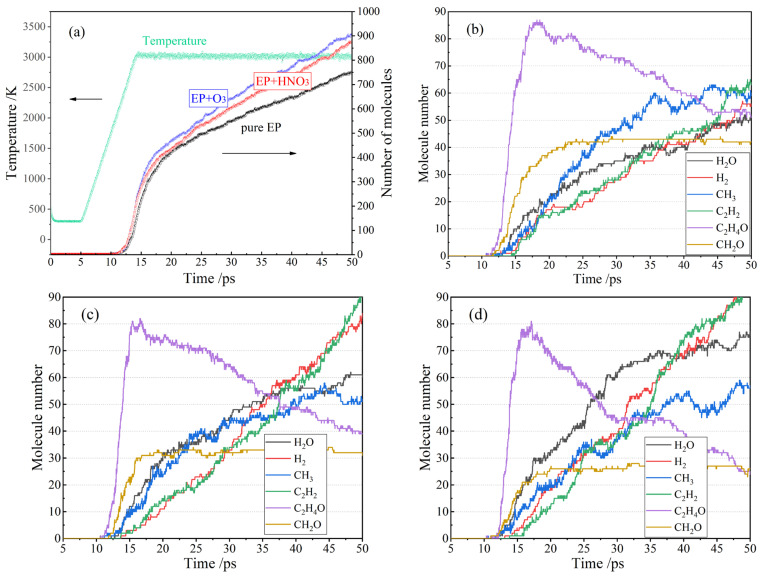
The amount of the produced small molecules increasing with time in reactive MD simulation process of crosslinked EP polymers under thermal impact: (**a**) temperature and total molecule number; (**b**) pure epoxy polymer; (**c**) containing nitric acid molecules; (**d**) containing ozone molecules.

Simulating the thermal effect caused by the electrical partial discharge and the chemical corrosion by nitric acid and ozone, the EP polymers can be decomposed into final products of fundamental molecules or radicals, which are mainly comprised of ethyne (C_2_H_2_), methylene radical (CH_3_), water (H_2_O), and hydrogen (H_2_), as shown in Figure 3. The simulated dynamic process of EP polymer decomposition indicates that the ether bonds (C-O-C) connecting benzene rings, C-C bonds adjoining carbonyl groups, and C-C bonds on both sides of the hydroxyl groups at different sites of EP molecular chains break to generate two major products of C_2_H_4_O and CH_2_O after ~17ps first, and the C_2_H_4_O is further decomposed into the final products of H_2_O, H_2_, CH_3,_ and C_2_H_2_. The amount of the final products continuously increases with time.

The initial temperature of EP polymer decomposition can be identified at the point that various small molecules start to appear in the simulation. The initiation temperature is significantly lower for impurity-containing EP polymers than that of the pure EP polymer. Nitric acid and ozone represent typical molecules generated by electric partial discharge, and their presence accelerates the synergetic thermal–electrical aging of EP materials through chemical decomposition. At the beginning of decomposition, when C_2_H_4_O and CH_2_O are generated, the two species of highly oxidizing impurities (HNO_3_ and O_3_) can evidently increase the amount of H_2_O and CH_3_, and somehow inhibit the generation of H_2_ and C_2_H_2_. Distinctively, both HNO_3_ and O_3_ impurities result in a remarkably higher yield of H_2_O and CH_3_ at the end of the MD process. The amounts of C_2_H_4_O and CH_2_O arising from the reactive dynamics process of impurity-containing EP polymers are higher than that of the pure EP polymer. CH_2_O is a final product, and the amount does not change by the end of the simulation. C_2_H_4_O is a characteristic intermediate product, which promptly approaches a maximum quantity with only a minority of final products, and then declines due to further decomposition. In the late stage of the thermal impact process, the maximum contents of C_2_H_4_O intermediate and CH_2_O final product are both reduced due to the existence of HNO_3_ or O_3_ impurity. It is therefore concluded that active molecular products generated by electric partial discharge, such as nitric acid and ozone, will aggravate the thermal-impacted decomposition of EP materials.

### 3.3. Chemical Bond Breaking

Reactive MD simulations show that active ions impacting on EP polymers at a high electric field will lead to considerable chemical decomposition and dehydrogenation. The kinetic energy acquired from the high electric field increases the collision frequency of active ions with EP molecular chains, through which the electric energy obtained by active ions is transformed into heat energy of EP polymers. This will cause a significant exacerbation in atomic vibrations (and thus a higher local temperature) and increase the probability of EP oxidation by active ions. Meanwhile, a higher electric field strength or chemical activity of ion adsorbates will aggravate EP polymer oxidization. The electric impact of negative active ions (O_3_^−^) gives rise to a graver chemical corrosion by dehydrogenation, primarily from hydroxyl, than that of NO_3_^+^. In addition, the various charged polar molecular groups generated in the decomposition and oxidization undergo a directional movement, further impacting the EP crosslinking structure under electric fields, which will in turn intensify the molecular structural degradation of EP polymers. It is thus suggested that the active ions generated from electrical partial discharge can directly enhance the oxidization of functional groups and the decomposition of molecular chains in EP polymers.

**Figure 4:**
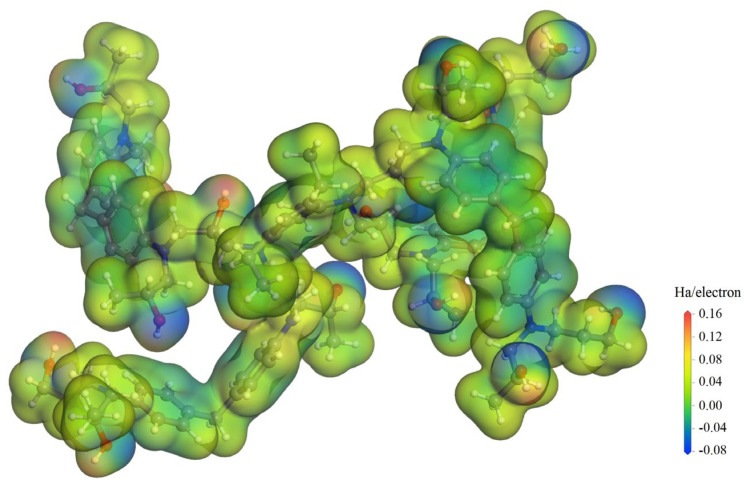
Electrostatic potential in TGDDM-DETDA crosslinked EP polymer, as described by color contours on electron density isosurface of 0.05 Å^−3^, in which the gray, blue, red, and white spheres denote carbon, nitrogen, oxygen, and hydrogen atoms, respectively.

Electrostatic potential distributions in the EP molecular model of crosslinking multiple TGDDM monomers with DETDA curing agent (representing the basic network units of crosslinked EP polymers) are calculated by all-electron numerical orbital first-principles schemes [31]. This is implemented by DMol3 code of Materials Studio package (BIOVIA, San Diego, CA, USA), as shown in Figure 4. The impacting on EP molecular chains by the positive H^+^ and negative (O_3_^−^ and NO_3_^−^) ions, which are derived from O_3_ or HNO_3_ ionization as described by reactive force field, are highly dependent of intrinsic electrostatic potentials of EP polymers, which is much higher than the externally applied electric field. This intrinsic electrostatic potential is higher (O/N atoms) and lower (C atoms adjoining O/N atoms) around the polar bonds of carbon nitrogen bridge bonds (-N-C-)/ether bonds (-C-O-C-)/alcohol group(C-O-C/C-OH), respectively. Under external electric fields, H^+^ and (O_3_^−^ + NO_3_^−^) ions are accelerated to impact preferentially onto the O/N atoms and the adjacent C atoms, respectively, leading to aggravations of atomic vibrations in these polar groups (local thermal effect), which will eventually cause the breakages of -C-O-C-, C-C bonds adjoining to hydroxy group, and C-N bonds at the crosslinking point of curing agent in EP polymers. The strong oxidation ability of O_3_ or NO_3_ accounts for the preferential oxidization of the hydroxyl groups into carbonyl groups (dehydrogenic reaction) before the local thermal effect initiates the chemical bond breaking of EP polymer backbone.

To this end, the polar bonds on the molecular chain of EP polymers inevitably give rise to higher and lower electrostatic potential regions than the nonpolar C-C backbone, which will become the focused areas of electrical or thermal impacts by active impurity ions, thus being vulnerable to decomposition aging from partial discharge under a high electric field. Therefore, abating the chemical components of forming polar bonds in epoxy resin, such as the ether bonds in epoxy monomer and the carbon nitrogen bridge bonds at crosslink joint of amine curing agent, can alleviate the decomposition aging of epoxy resin caused by the active impurities and thermal effect of partial discharge. A crosslinked EP polymer composed of the epoxy monomers with a lower number of ether bonds and the curing agent with fewer electronegative atoms at crosslink nodes is preferable to achieve a higher resistance to thermal-electrical decomposition aging or partial discharge aging.

Representative chemical bonding energies in the EP molecular backbone are calculated by the first-principles method, as shown in Table 1. The carbon bonds in -CH_2_-CHOH- and ether bonds (-C_6_H_4_-O-CH_2_-) show a higher bonding energy than that of carbon nitrogen bridge bonds (-NH-C-), in which the ether bonds connecting the benzene ring present the lowest bonding energy, and the average bond energy of the two C-C bonds adjoining hydroxyl group is reduced after hydroxyl group has been oxidized into carbonyl group. Thus, at the beginning of MD process, when active ions initiate EP polymer decomposition, the molecular chains will be firstly broken at the position of the C-O bonds connecting benzene group. However, due to the strong oxidability of active molecules and the high hydroxyl reductivity of EP polymers, a large number of hydroxyl groups is already oxidized to carbonyl groups before the molecular chains of EP polymer backbone are broken. Consequently, at the later stages in the reactive MD process of the EP polymeric model incorporating the ionization of active impurities, the molecular chain fracture occurs mainly at the C-C bonds adjoining the carbonyl groups generated from hydroxyl oxidation. Meanwhile, due to the intensified local thermal effect, the electric impact of active ions will also cause a minority of C-N bonds to break at the crosslinking points of curing agents despite their higher bonding energies. Since the reducibility of ammonia (dehydrogenation) in EP polymers is much lower than that of hydroxyl group, and the general epoxy resin materials have the largest number of hydroxyl groups, it can be rationally deduced that the molecular chain fracture of EP polymers under the electric impact of active ions will mainly occur on the C-C bonds on both sides of each hydroxyl group.

## 4. Conclusions

Crosslinked polymer models of EP materials, containing active molecular products derived from electrical partial discharges, are established to simulate their decomposition processes under thermal-electrical synergy conditions. Bond-boost acceleration method is employed for reactive molecular dynamics simulations to successfully establish the condensed model of 93% crosslinked EP polymer. The amount and species of decomposed molecular products are employed to study the decomposition temperature of the crosslinked molecular structure and evaluate the extent of decomposition. It is suggested that the high electric field in EP insulators cannot directly cause EP polymer decomposition, while the decomposition products acquiring kinetic energies from the high electric field will collide with EP molecular chains to promote a local thermal effect and thus aggravate the decomposition. Both nitric acid and ozone, which are generally engendered by partial discharge in EP materials, can exacerbate the thermal-impacted decomposition of the crosslinked EP polymer, as indicated by the decreased initial decomposition temperature from 1050 K to 940 K and 820 K, respectively. In consistence to the polar electrostatic potentials and lower bond energies from first-principles calculations, reactive MD dynamics simulations demonstrate that the ether bonds and carbon–nitrogen bridge bonds are vulnerable to the chemical corrosion of nitric acid and ozone and are preferentially broken by thermal impact for EP polymer decomposition. As described by a synergetic thermal-electrical decomposition process of EP polymer aging under partial discharges, the nitric acid and ozone generally produced by partial discharges are predicted to aggravate the thermal-impacted decomposition of EP materials under thermal effect of partial discharges.

## Figures and Tables

**Table 1:** First-principles bonding energies of representative C-C, C-O and N-C bonds in the molecular backbone of EP polymer (1 Ha = 27.2 eV).

Molecular Chain Energy/Ha	Broken Position	Broken Flakes	Flake Energy/Ha	Bonding Energy/Ha
EP unit −877.468	C-C bond1 adjoining Hydroxy -C_6_H_4_-O-CH_2_---CHOH-CH_2_-	1	−381.833	0.225997
		2	−495.409	
	C-C bond2 adjoining Hydroxy -C_6_H_4_-O-CH_2_-CHOH---CH_2_-	1	−495.409	0.198625
		2	−381.860	
	ether C-O bond1 -C_6_H_4_---O-CH_2_-CHOH-CH_2_-	1	−268.237	0.254078
		2	−608.976	
	ether C-O bond2 -C_6_H_4_-O---CH_2_-CHOH-CH_2_-	1	−342.955	0.185444
		2	−534.327	
EP Carboxide −876.279	carbonyl-adjoined C-C bond1 -C_6_H_4_-O-CH_2_-CO---CH_2_-	1	−494.229	0.190381
		2	−381.860	
	carbonyl-adjoined C-C bond2 -C_6_H_4_-O-CH_2_---CO-CH_2_-	1	−381.833	0.21185
		2	−494.235	
EP-curing unit −822.307	N bridging C-N bond1 -C_6_H_10_-NH---CH_2_-CHOH-	1	−271.809	0.226659
		2	−550.272	
	N bridging C-N bond2 -C_6_H_10_---NH-CH_2_-CHOH-	1	−326.677	0.232683
		2	−495.398	

## Data Availability

Theoretical results are available from the first author.
